# Supplementary material for: Vimentin filaments interact with the actin cortex in mitosis allowing normal cell division
Source: Nat Commun. 2019 Sep 13;10:4200. doi: 10.1038/s41467-019-12029-4 (PMC6744490; doi:10.1038/s41467-019-12029-4)
Supplement: Supplementary file 1 — Supplementary Information [file 41467_2019_12029_MOESM1_ESM.pdf]

Supplementary information for:

Vimentin filaments interact with the actin cortex in mitosis allowing normal cell division

Sofia Duarte, Álvaro Viedma-Poyatos, Elena Navarro-Carrasco, Alma E. Martínez, María A. Pajares, Dolores Pérez-Sala*

1. Supplementary figures

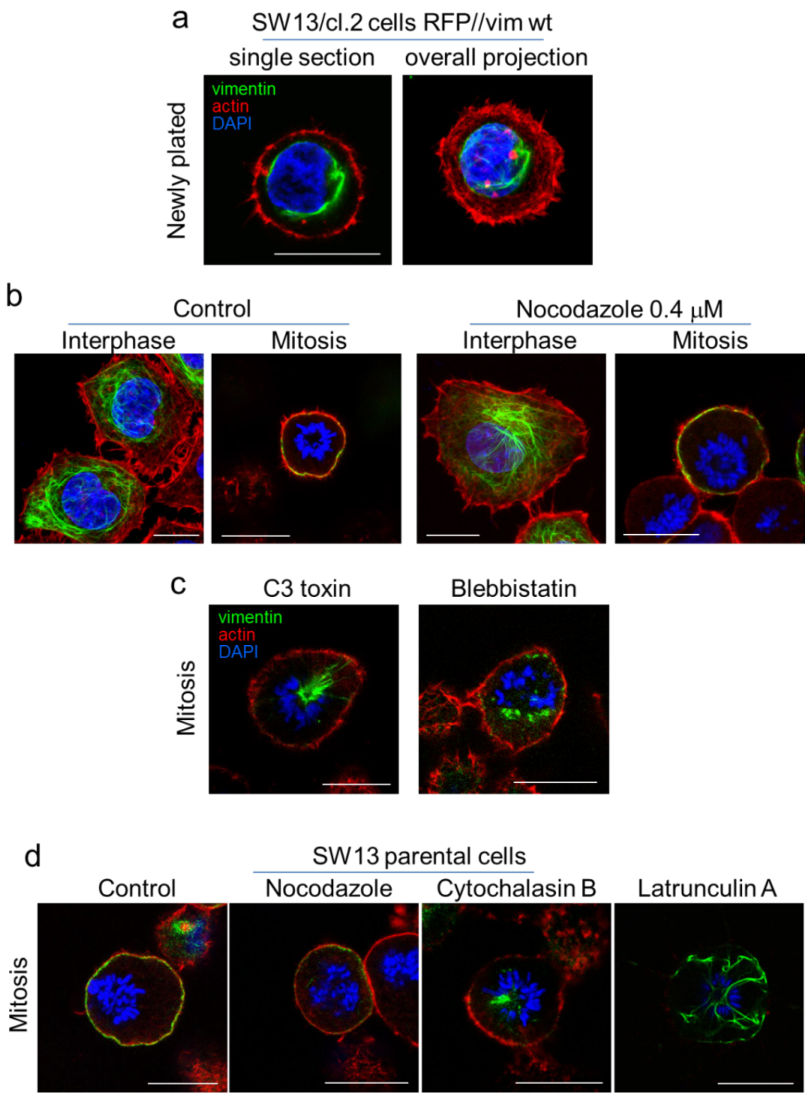

Supplementary Figure 1. **Effect of various approaches to modulate the cytoskeleton in SW13/cl.2 and parental SW13 cells.** (a) SW13/cl.2 cells stably transfected with RFP//vimentin wt (untagged vimentin wt) were detached by trypsinization and plated in a new dish. Cells were fixed 10 min after plating and the distribution of vimentin and f-actin was assessed by immunofluorescence and phalloidin staining, respectively. Nuclei were counterstained with DAPI. Left, single overlay; right, overall projection. (b and c) SW13/cl.2 cells stably transfected with RFP//vimentin wt were treated with vehicle (0.015% (v/v) DMSO) or a low concentration of nocodazole (0.4 μM) overnight in complete medium (b) or treated with 2 μg/ml C3 toxin for 3 h, or 20 μM blebbistatin for 1 h (c). The distribution of vimentin and f-actin in interphase and/or mitotic cells was monitored as above. (d) SW13 parental cells were treated with the indicated agents as detailed in the Materials and Methods section and processed as above. Images shown in (b) through (d) are single overlays. Scale bars, 20 μm.

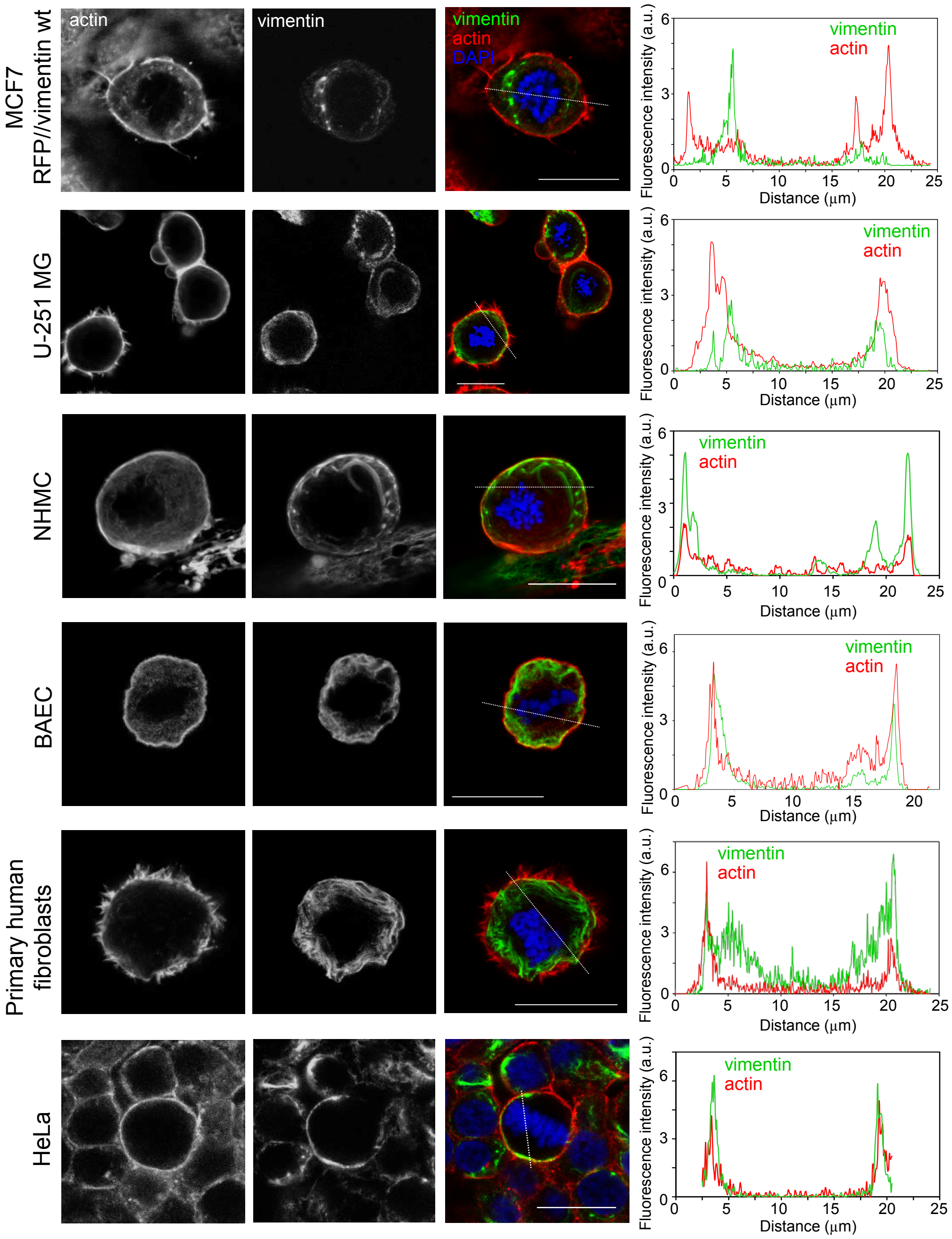

Supplementary Figure 2. **Distribution of vimentin in mitosis in several cell types.** The distribution of f-actin and vimentin in mitotic cells was assessed in several cell types expressing endogenous (U-251 MG, NHMC, BAEC, human dermal fibroblasts, HeLa) or transfected (MCF7) vimentin, as detailed in Fig. 4A. Single channel images from the overlays depicted in Fig. 4A, are shown. The fluorescence intensity profiles for vimentin and f-actin along the dotted lines drawn in the merged panels are shown at the right for every condition. Scale bars, 20 μm.

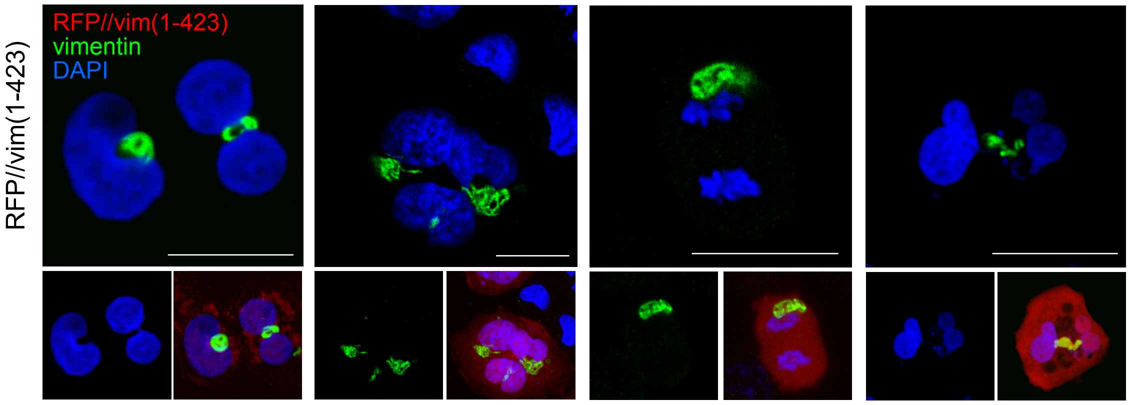

Supplementary Figure 3. **Additional images of the distribution of vimentin(1-423) in mitotic cells.** SW13/cl.2 cells were transfected with RFP//vimentin(1-423) and the distribution of vimentin(1-423) was observed by immunofluorescence. Single vimentin/DAPI overlays taken at mid-cell height are shown (upper panels). Lower panels depict overall projections for either DAPI or vimentin alone (left) or for the merge (right) of the three channels (vimentin, RFP as control of transfection, and DAPI). Scale bars, 20 μm.

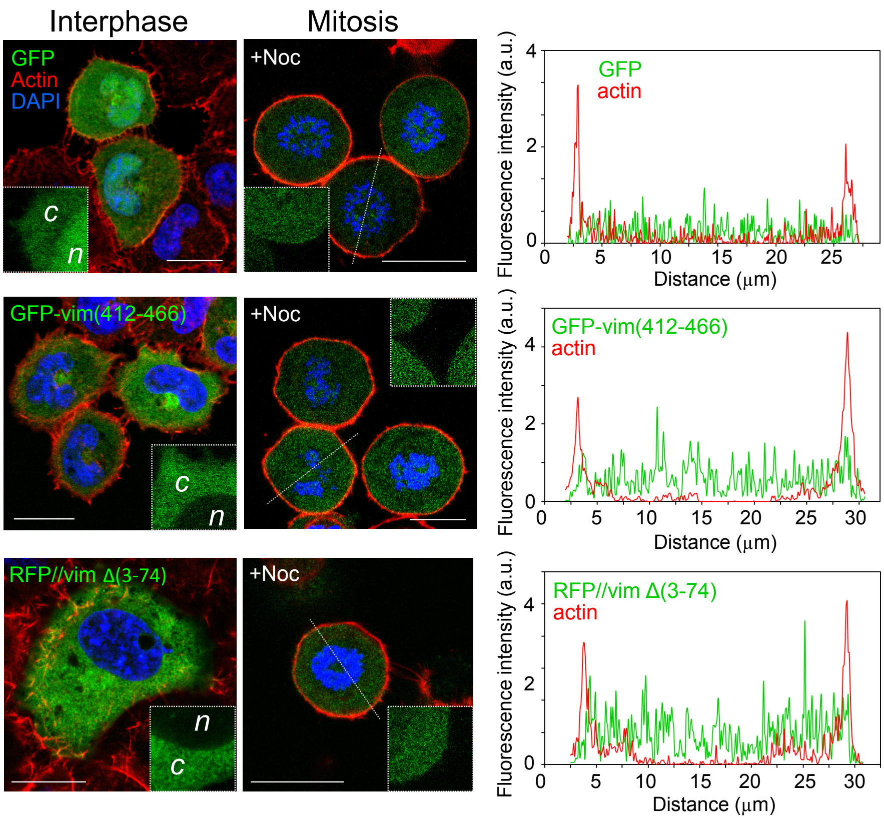

Supplementary Figure 4. **Localization of chimeric vimentin constructs.** SW13/cl.2 cells were transfected with the indicated plasmids and the localization of the resulting proteins and that of f-actin was assessed as above in interphase (left images) and dividing (right images) cells, after mild nococazole treatment. Single sections at mid-height of the cell are shown. Insets depict enlarged areas of interest of the green channel only to highlight the cytoplasm(*c*)-nuclear(*n*) boundary in interphase cells or the periphery of mitotic cells to show the cortical region. Note that in interphase cells GFP is diffuse throughout the cell whereas vimentin constructs remain in the cytoplasm. None of the constructs was enriched at the cell periphery. Fluorescence intensity profiles for vimentin and f-actin along the dotted lines in dividing cells are shown in the right panels. Scale bars, 20 μm.

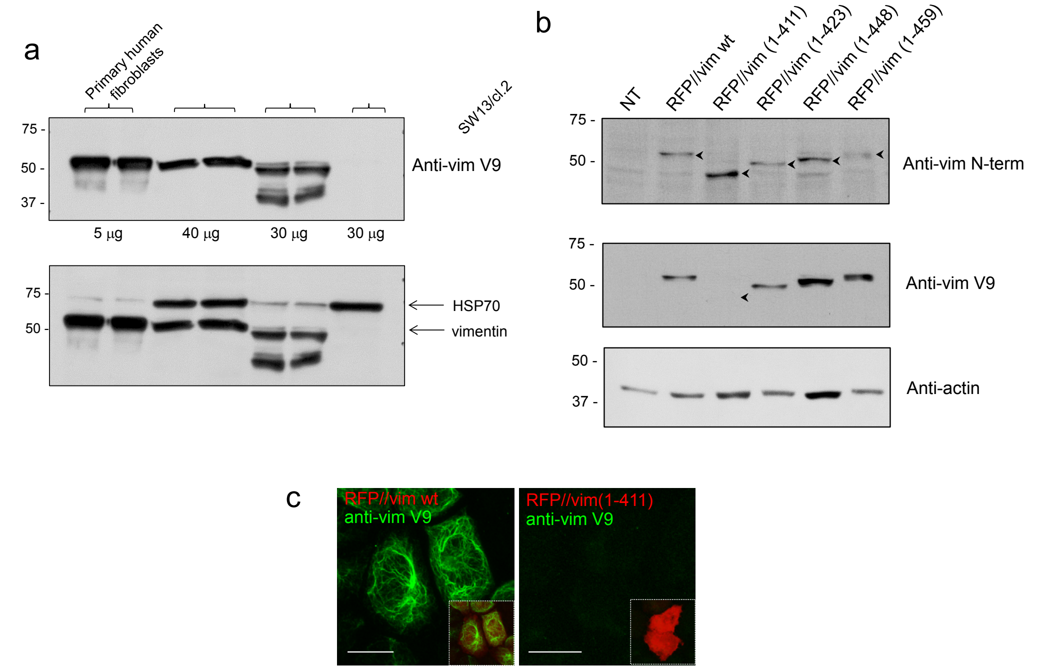

Supplementary Figure 5. **Levels, electrophoretical mobility and immunoreactivity of endogenous vimentin or expressed vimentin constructs in several cell types.** (a) Lysates from primary human fibroblasts, SW13/cl.2 cells stably transfected with RFP//vimentin wt, Vero cells or non-transfected SW13/cl.2 cells, containing the indicated amounts of total protein, were analyzed by SDS-PAGE and western blot with anti-vimentin antibody (upper panel). Subsequently, the same membrane was incubated with anti-Hsp70 antibody as a control (lower panel). (b) Immunoblot analysis of wild type or truncated vimentin constructs. SW13/cl.2 cells were transfected with the indicated constructs coding for vimentin wt or the various truncated forms. Cell lysates were analyzed by Western blot with antibodies against the N-terminus (upper panel) or the C-terminus of vimentin (middle panel, V9 monoclonal antibody). The position of the constructs is indicated by arrowheads. Note that the V9 clone does not recognize vimentin(1-411). The same membrane was incubated with anti-actin antibody as a control (lower panel). (c) The monoclonal anti-vimentin V9 antibody recognizes wt but not tailless vimentin by immunofluorescence. SW13/cl.2 cells were transfected with the indicated bicistronic constructs coding for vimentin wt of (1-411) and RFP as separate products. Vimentin was detected by immunofluorescence with Alexa488-conjugated anti-vimentin antibody, clone V9. Insets show overlays of the green (vimentin) and red (RFP) fluorescent signals. Bar, 20 µm.

2. Supplementary Tables

Supplementary Table 1: **Cell types used in the study**

**Cell types used in the study**	**Origin**	Endogenous vimentin	Cortical association of endogenous or transfected vimentin	Source	Independent authentication procedure
**1. Cancer cell lines**					
SW13/cl.2	Adrenal carcinoma	No	Yes	A Sarriá	
SW13	Adrenal carcinoma	Yes	Yes	ECACC	
MCF7	Breast carcinoma	No	No	ATCC	Short tandem repeat-PCR profiling (Secugen)
U-251 MG	Astrocytoma	Yes	Yes	ATCC	Short tandem repeat-PCR profiling (Secugen)
HAP1 vim (-)	Mielogenous leukemia	No	No	Horizon	
HeLa	Cervical carcinoma	Yes	Yes	ATCC	Short tandem repeat-PCR profiling (Secugen)
**2. Non-cancer cells**					
**2.1. Cell lines**					
Undifferentiated C2C12	Myoblasts	NA	Not explored	CIB-CSIC	Phenotype upon differentiation induced by horse serum; myogenin expression
Vero	Green monkey kidney	Yes	Yes	CIB-CSIC	Morphology
**2.2. Primary cultures**					
BAEC	Bovine aortic endothelial	Yes	Partial	Lonza	
Human dermal fibroblasts	Human dermis	Yes	Partial	Coriell	
Human mesangial cells	Human kidney	Yes	Yes	Clonetics	

Supplementary Table 2. **Constructs used in the study**

	Name	Abbreviation	Nature	Products expressed	Tag
1	pIRES-DsRed Express2	RFP//	Bicistronic	RFP	NA
2	pIRES-DsRed Express2-vimentin wt	RFP//vim wt	Bicistronic	RFP and vimentin aa 1 to 466 (separate products)	None
3	pIRES-DsRed Express2-vimentin(1-411)	RFP//vim(1-411)	Bicistronic	RFP and vimentin aa 1 to 411 (separate products)	None
4	pIRES-DsRed Express2-vimentin(1-423)	RFP//vim(1-423)	Bicistronic	RFP and vimentin aa 1 to 423 (separate products)	None
5	pIRES-DsRed Express2-vimentin(1-448)	RFP//vim(1-448)	Bicistronic	RFP and vimentin aa 1 to 448 (separate products)	None
6	pIRES-DsRed Express2-vimentin(1-459)	RFP//vim(1-459)	Bicistronic	RFP and vimentin aa 1 to 459 (separate products)	None
7	pIRES-DsRed Express2-vimentin Δ(3-74)	RFP//vim Δ(3-74)	Bicistronic	RFP and vimentin aa (1-2)(75-466) (separate products)	None
8	pEGFP-C1	GFP	Vector	GFP	NA
9	pEGFP-C1-vimentin wt	GFP-vim wt	Fusion	GFP fused to vimentin wt	GFP
10	pEGFP-C1-vimentin(1-411)	GFP-vim(1-411)	Fusion	GFP fused to aa 1 to 411 of vimentin	GFP
11	pEGFP-C1-vimentin(1-423)	GFP-vim(1-423)	Fusion	GFP fused to aa 1 to 423 of vimentin	GFP
12	pEGFP-C1-vimentin(1-448)	GFP-vim(1-448)	Fusion	GFP fused to aa 1 to 448 of vimentin	GFP
13	pEGFP-C1-vimentin(1-459)	GFP-vim(1-459)	Fusion	GFP fused to aa 1 to 459 of vimentin	GFP
14	pEGFP-C1-vimentin C328S	GFP-vim C328S	Fusion	GFP fused to vimentin C328S	GFP
15	pEGFP-C1-vimentin G452V	GFP-vim G452V	Fusion	GFP fused to vimentin G452V	GFP
16	pEGFP-C1-vimentin(412-466)	GFP-vim(412-466)	Fusion	GFP fused to aa 412 to 466 of vimentin	GFP
17	mCherry-vimentin wt	mCherry-vimentin wt	Fusion	mCherry-vimentin wt	mCherry
18	pECFP-C1-Lamin A	CFP-Lamin A	Fusion	CFP-Lamin A	CFP
19	pcDNA3/GFP-PR	GFP-PR	Fusion	GFP-HIV type I protease	GFP

Supplementary Table 3. **Sequences of oligonucleotides used for mutagenesis**

Protein	Accesion No.	Mutant construct	Oligonucleotide sequence*
Human vimentin	NP_003371	Vimentin(1-411)	5'-GGCGAGGAGAGCAGGATTT**AA**CTGCCTCTTCCAAACTTTTCC-3'
"	"	Vimentin(1-423)	5'-CCTCCCTGAACCTG**TA**GGAAACTAATCTGGATTCACTCC-3'
"	"	Vimentin(1-448)	5'-GGACACTTCTGATTAAGACGGTTGAA**TGA**AGAGATGGACAGGTTATC-3'
"	"	Vimentin(1-459)	5'-GGTTATCAACGAAACTTCT**T**AGCATCACGATGACC-3'
"	"	Vimentin EcoRI(1692)	5’-GGCGAGGAGAGCAGGA**A**TTCTCTGCCTCTTCCAAACTTTTCC-3’
"	"	Vimentin SmaI(471)	5’-CGAATTCCATGTCC**C**CC**G**GGGTGCGGCTCC-3’
"	"	Vimentin G452V	5’-CGGTTGAAACTAGAGATG**T**ACAGGTTATCAACGAAACTTCTC-3’

*Nucleotide changed appear in bold
