# Supplementary material for: Vimentin filaments interact with the actin cortex in mitosis allowing normal cell division
Source: Nat Commun. 2019 Sep 13;10:4200. doi: 10.1038/s41467-019-12029-4 (PMC6744490; doi:10.1038/s41467-019-12029-4)
Supplement: Supplementary file 3 — Description of Additional Supplementary Files [file 41467_2019_12029_MOESM3_ESM.docx]

**Description of Additional Supplementary Files**

**File Name: Supplementary Movie 1**

**Description:** Mitosis of SW13 cells expressing RFP//vimentin wt (1). Cells were processed as detailed in the legend of Fig. 2.

**File Name: Supplementary Movie 2**

**Description:** Mitosis of SW13 cells expressing RFP//vimentin wt (2). Cells were processed as detailed in the legend of Fig. 2.

**File Name: Supplementary Movie 3**

**Description:** Mitosis of SW13 cells expressing RFP//vimentin(1-411) ending in mitotic catastrophe. Cells were processed as detailed in the legend of Fig. 2.

**File Name: Supplementary Movie 4**

**Description:** Mitosis of SW13 cells expressing RFP//vimentin(1-411) ending in asymmetric partition of vimentin. Cells were processed as detailed in the legend of Fig. 2.

**File Name: Supplementary Movie 5**

**Description:** 3D-reconstruction of the bottom half of the cell shown in Fig. 5C.

**File Name: Supplementary Movie 6**

**Description:** 3D-reconstruction of vimentin-positive cell shown in Fig. 6B.

**File Name: Supplementary Movie 7**

**Description:** 3D-reconstruction of vimentin-negative cell shown in Fig. 6B.

**File Name: Supplementary Movie 8**

**Description:** Mitosis of SW13 cells expressing RFP//vimentin(1-423) ending in asymmetric partition of vimentin. Cells were processed as detailed in the legend of Fig. 7.

**File Name: Supplementary Movie 9**

**Description:** Mitosis of SW13 cells expressing RFP//vimentin(1-423) ending in mitotic catastrophe. Cells were processed as detailed in the legend of Fig. 7.

**File Name: Supplementary Movie 10**

**Description:** Mitosis of SW13 cells expressing RFP//vimentin(1-448) ending in mitotic catastrophe. Cells were processed as detailed in the legend of Fig. 7.

**File Name: Supplementary Movie 11**

**Description:** Mitosis of SW13 cells expressing RFP//vimentin(1-448) ending in asymmetric partition of vimentin. Cells were processed as detailed in the legend of Fig. 7.

**File Name: Supplementary Movie 12**

**Description:** Mitosis of SW13 cells expressing RFP//vimentin(1-459) ending in normal division. Cells were processed as detailed in the legend of Fig. 7.

**File Name: Supplementary Movie 13**

**Description:** Mitosis of SW13 cells expressing RFP//vimentin(1-459) ending in asymmetric partition of vimentin. Cells were processed as detailed in the legend of Fig. 7.
